# Reported Needs and Depressive Symptoms Among Older Adults Entering Long-Term Services and Supports

**DOI:** 10.1093/geroni/igaa021

**Published:** 2020-06-09

**Authors:** Eleanor Rivera, Karen B Hirschman, Mary D Naylor

**Affiliations:** NewCourtland Center for Transitions and Health, University of Pennsylvania School of Nursing, Philadelphia

**Keywords:** Transportation, Equipment and supplies, Social participation, Depression, Residential facilities, Community health services, Aged

## Abstract

**Background and Objectives:**

Long-term services and supports (LTSS) are vital for older adults with physical and cognitive disabilities. LTSS can be provided in settings such as nursing homes, assisted living, or via community-based services. During the transition to LTSS, older adults are at risk of increased depressive symptoms. In addition, older adults may identify unmet needs despite having access to new LTSS resources. The goal of this study was to examine the factors associated with increased depressive symptoms among a pool of older adults, with a focus on change in reported needs after starting LTSS.

**Research Design and Methods:**

This cross-sectional analysis of a cohort study included 352 older adults new to LTSS (R01AG025524). The outcome of depressive symptoms was measured using the Geriatric Depression Scale—Short Form. Reported needs included supportive equipment, devices, transportation, and social activities. Bivariate and linear regression modeling using change in needs 3 months later were performed.

**Results:**

Depressive symptoms were present among 40% of the LTSS recipients at enrollment and 3 months. At baseline, 29% of LTSS recipients reported a need for supportive equipment, 30% for transportation, and 23% for social activities. After 3 months, an average of 12% of LTSS recipients’ needs were met, 13% of LTSS recipients’ needs persisted, and 11% of LTSS recipients reported new needs. Depressive symptoms 3 months later were higher for those who reported persistent unmet needs compared with those who reported no needs at all, controlling for functional status and LTSS type.

**Discussion and Implications:**

The transition to LTSS is a vulnerable time for older adults. Assessing the need for equipment, transportation, and social activities during this period may identify opportunities to improve the lives and emotional status of this population.

**Translational Significance:** Depression is common among older adults transitioning to long-term services and supports (LTSS). The need for equipment, transportation, and access to social activities all affect LTSS recipients’ emotional status. A focus on assessing these needs more thoroughly from the start of LTSS and trying to meet those needs may have a positive impact on the mental health of older adults.

## Background and Objectives

The population of the United States continues to shift toward an older average age with more than 52 million people aged 65 or older as of 2018 (Population Reference Bureau, 2019). With the expected doubling of individuals 65 and older to 95 million by 2060 comes a corresponding increase in the need for long-term services and supports (LTSS) (Population Reference Bureau, 2019). LTSS are vital services for older adults who require additional support with daily tasks (such as eating and bathing) in their own home and other settings such as nursing homes (NHs) and assisted living communities (ALC) (Medicaid and CHIP Payment and Access Commission, 2017). These services are comprehensive and could include providing meals, administering medications, or access to facilities with enhancements for those with limited mobility. Ten percent of all Medicaid spending annually is on LTSS for older adults, equaling more than $50 billion (Centers for Medicare & Medicaid Services, 2016; Medicaid and CHIP Payment and Access Commission, 2017).

Depressive symptoms are common in both institution-based (24%–37%) and home-based LTSS settings (28%) (Li et al., 2019; Pepin, Leggett, Sonnega, & Assari, 2017; Seitz, Purandare, & Conn, 2010; Vouri, Crist, Sutcliffe, & Austin, 2015; Watson, Garrett, Sloane, Gruber-Baldini, & Zimmerman, 2003) and are related to older adults’ feelings of belonging in their environment and the level of support they receive (McLaren, Turner, Gomez, McLachlan, & Gibbs, 2013; Park, Smith, Dunkle, Ingersoll-Dayton, & Antonucci, 2019). The transition to LTSS is often a result of acute events such as hospitalizations, falls, or exacerbations of chronic conditions, potentially introducing or intensifying stressors that increase risk of depressive symptoms for older adults (Pot, Deeg, Twisk, Beekman, & Zarit, 2005; Robison, Shugrue, Porter, Fortinsky, & Curry, 2012; Ulbricht, Rothschild, Hunnicutt, & Lapane, 2017). During episodes of acute illness, depressive symptoms among older adults are associated with increased risk of suicide, all-cause mortality, increased use of health care services, and worsening chronic disease status and are especially high in older adults in LTSS settings (Alexopoulos, 2005; Blazer, 2003; Institute of Medicine, 2012).

Throughout the vulnerable transition to receiving LTSS, a potential contributor to increased depressive symptoms for older adults may be unmet needs (Xiang, An, & Heinemann, 2018). The need for supportive equipment (such as wheelchairs, walkers, or grab bars) is common among older adults and may be difficult to acquire or afford. Not fulfilling this equipment need can lead to negative experiences such as poor hygiene, social isolation, and increased rates of falls (Willink et al., 2019). Similarly, there is an associated link between driving cessation and increased depressive symptoms among older adults which may be due to the new need for transportation provided by other individuals or services (Chihuri et al., 2016; Fonda, Wallace, & Herzog, 2001). Also, access to and participation in social activities have been found to slow functional decline and improve depressive symptoms in older adults (Buchman et al., 2009; Forsman, Nordmyr, & Wahlbeck, 2011). The transition to receiving LTSS is the optimal time to assess both depressive symptoms and the need for equipment, transportation, and social activities, but follow-up evaluation to determine whether these needs are met also is critical. To date, the relationship between depressive symptoms and reported needs during this early transition time has not been studied in this population.

The goal of this study was to examine the factors associated with the presence of depressive symptoms among a group of older adults in the early stages of transition to LTSS settings: approximately 3 months after the start of receiving LTSS in an NH, ALC, or in the community/in their home. Our hypothesis was that, among this population of older adults, unmet needs (supportive equipment, transportation, and social activities) will be an important predictor of higher levels of depressive symptoms 3 months later.

## Research Design and Methods

### Data Set

Data used in these analyses came from the “Health Related Quality of Life: Elders in Long Term Care” National Institutes of Health/National Institute on Aging funded (R01AG025524) longitudinal cohort study of older adults new to LTSS (Naylor et al., 2016). A total of 11 organizations in New Jersey, Pennsylvania, and New York participated: 24 NHs, 29 ALCs, and 5 home- and community-based services (HCBS) programs. The parent study enrolled 470 older adults (aged 60 and older) 2007–2010 (Naylor et al., 2016; Zubritsky et al., 2016) and followed participants for 2 years (through 2012). Older adults were eligible to enroll in the parent study if they were new to LTSS (within approximately 60 days from the start of services), planning to continue with the services long term, spoke English or Spanish, scored 12 or higher on the Mini-Mental State Examination (MMSE) (Folstein, Folstein, & McHugh, 1975), and were not considered terminally ill (e.g., <6 months to live). Individuals scoring between 12 and 22 on the MMSE (indicating some level of cognitive impairment) were asked to provide consent to participate and required a legally responsible party to also provide informed consent to let their family member participate (Naylor et al., 2016; Zubritsky et al., 2016).

Participants were interviewed at enrollment and then every 3 months through 24 months. The overall categories of data collected were: objective measures of health status, demographic information, cognitive status, symptom status, functional status, social support, general health, well-being, and perceived quality of life (Zubritsky et al., 2016). Interviews with participants were conducted face-to-face for self-reported responses for a majority of study variables. Data were also abstracted from chart reviews for all study participants. Additionally, some data were collected from individuals familiar with the participants for those with MMSE scores less than 23. For this analysis, the only variable that was collected in this manner was the activities of daily living (described in *Functional characteristics*). Demographic measures were collected only at baseline and all other data were collected at each 3-month time point.

Data for this study were restricted to individuals with complete outcome data at both baseline and 3 months (excluding *n* = 86). Participants who moved from one LTSS setting to another within the first 3 months were also excluded (*n* = 11) in order to keep the type of LTSS constant from enrollment to first follow-up. Those who scored less than 12 on the MMSE at the 3-month time point (*n* = 7) were excluded to harmonize the sample to the initial inclusion criteria. Finally, participants who did not have data for at least two of the three reported needs (the primary independent variables—supportive equipment, transportation, and social activities, described later) were excluded (*n* = 14). With these restrictions, 352 of the 470 original participants were included.

### Outcome

Depressive symptoms were measured using the Geriatric Depression Scale—Short Form (GDS-SF), where higher scores indicate more depressive symptoms (Conradsson et al., 2013; Sheikh & Yesavage, 1986; Yesavage, 1988; Yesavage et al., 1982). This instrument has 15 items with yes/no response options. Scores of 0–4 indicate a normal emotional state, scores of 5–10 are suggestive of mild to moderate depression, and scores of 10–15 are suggestive of severe depression (Burke, Nitcher, Roccaforte, & Wengel, 1992; Parmelee, Katz, & Lawton, 1989). The reliability and validity of this instrument have been tested extensively, including with older adults who have mild to moderate cognitive impairment (Conradsson et al., 2013; Lach, Chang, & Edwards, 2010).

### Covariates

All data used in this study were collected either during the initial baseline interview or the 3-month follow-up interview, with some LTSS recipient clinical characteristics confirmed or collected from a medical record review.

#### Demographic and individual characteristics

Demographic variables and covariates collected at baseline included sex (male/female), age (continuous), race (White, Black or African American, Asian, Native Hawaiian or Pacific Islander, American Indian or Alaskan Native, and more than one race), ethnicity (Hispanic/non-Hispanic), LTSS type (assisted living facility, NH, or HCBS), and number of comorbid conditions.

#### Needs

Several categories of perceived need (i.e., supportive equipment, transportation, and social activities) were asked about using a yes (=1) or no (=0) question format. Equipment needs were assessed with, “Do you need any equipment or aids that you currently do not have?” Transportation needs were assessed with, “Do you feel you need transportation more often than it is available to you now for appointments, visiting, social events, etc.?” Social activity needs were assessed with, “Do you feel there are enough activities that you like available to you now?” The valence of the response for social activity was switched to correspond with the other need variables, with yes responses indicating no need (=0) and no responses indicating need (=1). Categorical need change variables were created by comparing the answer to the need questions at baseline to the 3-month response. The answers were categorized as “no need at all” for those who reported no need at either time point, “need persists” for those who reported a need at both time points, “new need” for those who reported no need at baseline and a need 3 months later, and “no longer reported” for those who reported a need at baseline and no need 3 months later.

#### Functional characteristics

Cognitive status was measured using the 30-item MMSE score, where lower scores indicate greater cognitive impairment (Folstein et al., 1975). Physical function was measured using the 6-item Katz Basic Activities of Daily Living (BADL) scale, where lower scores indicate a greater level of dependence (Katz & Akpom, 1976). For participants with cognitive impairment (MMSE <23), the Katz BADL tool was completed by a caregiver or LTSS staff member familiar with the day-to-day care needs of the participant.

### Analyses

Statistical analyses included a comparison of the subset to the full data set (chi-square and *t* tests), descriptive statistics of the subsample, and bivariate and linear regression modeling of the outcome. There is a known confounding between LTSS type and race and ethnicity in this data set (Naylor et al., 2016). Therefore to control for confounding, all analyses include LTSS type as an independent variable and exclude race (ALC: White 92.9%, non-White 7.1%; NH: White 34.3%, non-White 65.7%; HCBS: White 27.9%, non-White 72.1%) and ethnicity (ALC: Hispanic 1.6%, non-Hispanic 98.4%; NH: Hispanic 2.9%, non-Hispanic 97.1%; HCBS: Hispanic 48.8%, non-Hispanic 51.2%) for the subset of 352 participants.

Regression models were built using a stepwise procedure. First, all covariates were regressed on the outcome of depression separately. Next, any covariates with a *p* value of .20 or less were included in a full model (Vittinghoff, McCulloch, & Glidden, 2005). Next, variables with *p* values greater than .05 were removed one at a time from the model until all variables were statistically significant at *p* ≤ .05 (Vittinghoff et al., 2005). Model fit testing was conducted using Akaike information criterion (AIC) and Bayesian information criterion (BIC) fit criteria. All models were run with and without a log-transformed outcome variable as a sensitivity analysis due to the right-sided skew of the GDS variable. The findings did not change. Nontransformed models are presented for ease of interpretation. All analyses were performed using STATA version 13 (StataCorp., 2017).

## Results

### Sample Characteristics

Descriptive statistics and baseline values for the subset sample (*n* = 352) are presented in Table 1. The majority of the sample was female (71%, 249/352), White (53%, 186/352), and on average 81 years old (range: 60–98 years). Of note, more than 30% (121/352) of the sample had some level of cognitive impairment (mild, MMSE 20–23: 68/352; moderate, MMSE 12–19: 53/352). Similarly, nearly 40% (141/352) reported depressive symptoms (mild to moderate, GDS 5–10: 125/352; severe, GDS 10–15: 16/352). There were no statistically significant differences between baseline and 3 months for the average number of comorbid conditions, cognitive status (MMSE scores), functional status (ADL scores), or depressive symptoms (GDS scores).

**Table 1.** Descriptive Statistics and Characteristics of the Sample at 3 Months After Baseline (*n* = 352)

Variable	*n* (%) or Mean ± *SD* (range)
Sex (female)	249 (71.7%)
Age	81.0 ± 8.6 (60–98)
Race	
White	186 (53.1%)
Black or African American	117 (33.4%)
Asian	2 (0.6%)
Native Hawaiian or Pacific Islander	3 (0.9%)
American Indian or Alaskan Native	4 (1.1%)
More than one race	38 (10.9%)
Ethnicity (Hispanic)	65 (18.5%)
LTSS type	
Assisted living facility	126 (35.8%)
Nursing home	103 (29.3%)
Home- and community-based services	123 (34.9%)
Comorbid conditions (number of)	8.6 ± 3.9 (1–27)
Mini-Mental State Examination (score range)	24.2 ± 4.3 (12–30)
Cognitively intact (24–30)	214 (60.8%)
Mildly impaired (20–23)	68 (19.3%)
Moderately impaired (12–19)	53 (15.1%)
Missing	17 (4.8%)
Katz—Activities of Daily Living score	4.6 ± 1.7 (0–6)
Geriatric Depression Scale—Short Form	4.2 ± 3.1 (0–13)
Normal (0–4)	211 (59.9%)
Mild to moderate depression (5–10)	125 (35.5%)
Severe depression (11–15)	16 (4.6%)

*Note:* The number of comorbid conditions, Mini-Mental State Examination scores, Activities of Daily Living scores, and Geriatric Depression Scale scores are the values collected at 3 months; all other data were collected at baseline; LTSS = long-term services and supports.

This subset (*n* = 352) was assessed for differences in sex, age, race, ethnicity, and LTSS facility type from the full data set at baseline (*n* = 470). Chi-square and *t* tests were performed between the full parent data set and the subset for these variables. Only LTSS was statistically significantly different, with a greater number of NH residents having missing data at baseline on variables of interest in this study (55/158, 35%) compared with LTSS recipients in assisted living (30/156, 19%) or HCBS (33/156, 21%) (χ ^2^ = 12.07, *p* = .002).

### Perceived Need

Baseline levels of perceived need varied (Table 2), with the highest reported needs at baseline being transportation (30%, 104/352) and supportive equipment (29%, 101/352) and fewer LTSS recipients reporting a need for social activities (23%, 82/352). The perceived need for transportation and supportive equipment declined 3 months later, decreasing 2.3% and 4.8%, respectively, whereas the need for social activities increased 3 months later (+1.7%). At baseline, 5.7% of LTSS recipients reported all three needs, 13.9% reported two needs, 29.3% reported one need, and 28.6% reported no needs. Three months later, 2.6% reported all three needs, 17.9% reported two needs, 24.2% reported one need, and 37.8% reported no needs. Data were missing for one or more needs at baseline (12.5%) and at the 3-month time point (17.6%).

**Table 2.** Long-Term Services and Supports Recipient Perceived Needs at Baseline and 3 Months Later

	Reporting need, *n* (%)		Change in perceived need at 3 months, *n* (%)				
	Baseline	3 months	Need persists	No longer reported	New need	No need at all	Missing
Transportation			52 (14.8)	42 (11.9)	40 (11.4)	164 (46.6)	54 (15.3)
Need	104 (29.5)	96 (27.2)					
No need	222 (63.1)	216 (61.4)					
Missing	26 (7.4)	40 (11.4)					
Supportive equipment			54 (15.3)	45 (12.8)	29 (8.2)	216 (61.4)	8 (2.3)
Need	101 (28.7)	84 (23.9)					
No need	246 (69.9)	265 (75.3)					
Missing	5 (1.4)	3 (0.9)					
Social activities			(10.2)	(10.2)	46 (13.1)	200 (56.8)	34 (9.7)
Need	82 (23.3)	88 (25.0)					
No need	252 (71.6)	245 (69.6)					
Missing	18 (5.1)	19 (5.4)					

A majority of the participants reported having none of these needs at either time point (transportation 47%, equipment 61%, and social activities 57%). The need for transportation, equipment, and social activities was consistently identified as a persistent need at both baseline and 3 months later by 15%, 15%, and 10% of respondents, respectively. Slightly smaller percentages of these needs at baseline were no longer reported 3 months later (transportation 12%, equipment 13%, and social activities 10%). Between 8% and 13% of the respondents reported a new need after 3 months that was not previously reported at baseline (transportation 11%, equipment 8%, and social activities 13%).

### Bivariate Analyses

Individual bivariate linear regression models with the outcome of interest, depressive symptoms, were generated for each of the covariates listed in Table 1 (sex, age, LTSS type, number of comorbid conditions, MMSE scores, and ADL scores) and each of the three need variables (equipment, transportation, and social activities). Bivariate regression models revealed several variables that were significant at *p* ≤ .20 and were used in building multivariable models: age (*p* = .136), LTSS type (HCBS vs. ALC, *p* < .0001; NH, *p* = .004), ADLs (*p* < .0001), equipment need (no need at all vs. need persists, *p* = .002; new need, *p* = .146; no longer needed, *p* = .015), transportation need (no need at all vs. need persists, *p* = .003), and social activities need (no need at all vs. need persists, *p* = .010; no longer needed, *p* = .034). The variables that did not meet the *p* ≤ .20 threshold and were not included in the multivariable model building were sex (*p* = .680), number of comorbid conditions (*p* = .632), MMSE score (*p* = .818), transportation needs (no need at all vs. new need, *p* = .215; no longer needed, *p* = .469), and social activities needs (no need at all vs new need, *p* = 0.435).

### Multivariable Analyses

Multivariable linear regression models were built to examine the impact of reported needs on depressive symptoms (GDS-SF scores). Three separate multivariable models were generated for each of the need variables including all variables significant at *p* ≤ .20 in bivariate analyses. All covariates entered into the models remained significant at *p* ≤ .05 with the exception of age, which fell out of all three models. Table 3 presents the results of the three multivariable regression models. All three models showed that persistent perceived needs, more BADL deficits, and living in the community receiving LTSS were associated with higher numbers of reported depressive symptoms. After controlling for all other covariates in the model, LTSS type was the strongest predictor of depressive symptoms overall, with the assisted living and NH settings both having lower depressive scores than home- and community-based settings in all three models (HCBS vs ALC: equipment and transportation needs models, *B* = −1.33, *p* = .001; social activities need model, *B* = −1.38, *p* = .001; HCBS vs NH: equipment and social activities needs models, *B* = −1.64, *p* < .001; transportation need model, *B* = −1.69, *p* < .001). Higher ADL scores also predicted lower depression scores in all three models (equipment need model, *B* = −0.40, *p* < .001; transportation need model, *B* = −0.42, *p* < .001; social activities need model, *B *= −0.44, *p* < .001). For the need variables, having a persistent need versus no need at all predicted higher depression scores (equipment, *B* = 1.01, *p* = .037; transportation, *B* = 1.19, *p* = .015; social activities, *B* = 1.11, *p* = .040).

**Table 3.** Multivariable Linear Regression Models of LTSS Recipients’ Depressive Symptoms at the 3-Month Time Point

	Equipment needs (*n* = 328)			Transportation needs (*n* = 292)			Social activities needs (*n* = 304)		
	*B*	95% CI	*p* Value	*B*	95% CI	*p* Value	*B*	95% CI	*p* Value
Need change									
No need at all		(ref.)			(ref.)			(ref.)	
Need persists	1.01	0.06, 1.96	.037	1.19	0.24, 2.15	.015	1.11	0.05, 2.17	.040
New need	0.57	−0.58, 1.72	.332	0.77	−0.24, 1.78	.135	0.49	−0.43, 1.41	.297
No longer needed	0.94	−0.05, 1.93	.062	0.21	−0.80, 1.23	.683	0.57	−0.52, 1.66	.303
Katz ADL score	−0.40	−0.60, −0.20	<.001	−0.42	−0.63, −0.21	<.001	−0.44	−0.64, −0.23	<.001
LTSS type									
HCBS		(ref.)			(ref.)			(ref.)	
AL	−1.33	−2.13, −0.54	.001	−1.33	−2.13, −0.54	.001	−1.38	−2.17, −0.58	.001
NH	−1.64	−2.47, −0.83	<.001	−1.69	−2.55, −0.83	<.001	−1.64	−2.49, −0.79	<.001
Adjusted *r*^2^	0.1113			0.1129			0.1111		

*Note:* ADL = activities of daily living; NH = nursing home; AL = assisted living; HCBS = home- and community-based services; *B* = beta coefficient; CI = confidence interval; LTSS = long-term services and supports.

We also assessed the impact of the other covariates that are commonly found to be associated with depressive symptoms in other research with this population (Dmitrieva et al., 2015; Parajuli, Berish, & Jao, 2019). Each of the models in Table 3 was compared with a model containing all of the other potential variables (sex, age, number of comorbid conditions, and MMSE scores) using AIC and BIC fit criteria. For all three needs variables, the AIC and BIC values were lower for the nested models, indicating that the nested models (those presented in Table 3) are a better fit than the full models that include all variables.

## Discussion and Implications

This study identified important relationships in the perceived needs of older adults and depressive symptoms within approximately 60 days of the start of LTSS and the 3 months following that baseline. Given the findings, among all three needs, the depression scores for LTSS recipients who had their needs met did not differ significantly from those who had no needs at all, though the coefficients indicate slightly higher GDS scores. Among LTSS recipients who had persistent needs, the GDS scores were statistically significantly higher than those who had no needs at all. Finally, LTSS recipients who reported new needs did not differ significantly from those who had no needs at all, though the coefficients again indicate slightly higher GDS scores. This may indicate that new needs emerging during the short 3-month time frame when this question was asked had not yet had an impact on depressive symptoms. To date, no other studies have analyzed the relationship between depressive symptoms and reported needs of older adults during the period of early transition into LTSS. By the 3-month time point, there appears to be some improvement in the reported needs (e.g., equipment and transportation), indicating that entering into LTSS may be providing opportunities for older adults’ needs to be assessed and addressed by the new resources and support. However, 8%–13% of LTSS recipients identified new needs at the 3-month interview, highlighting the necessity for ongoing assessment to fully capture the evolving needs of older adults such as new functional limitations or possibly internal reevaluation of what is possible to ask of their LTSS setting.

Given the well-documented relationship between depressive symptoms and poor health outcomes (Ho et al., 2014; Rackley & Bostwick, 2012), meeting these needs could be a key strategy to improve the health and quality of life of older adults in LTSS settings. Recent changes to the Medicare Advantage plans have expanded coverage for supplemental benefit services that are not explicitly health-related, as long as those services could be reasonably expected to improve health (Anne Tumlinson Innovations & Long-Term Quality Alliance, 2019). Although these expansions in coverage are new and the impact not yet evaluated, it is possible that giving LTSS providers the opportunity to bill for these types of services could provide financial support to meet the needs of older adults in LTSS settings. We would advocate for government policy and reimbursement changes like these that would support an LTSS provider’s ability to meet needs that may not always been in their current scope. The ability to meet the perceived needs of LTSS recipients using nonpharmacological strategies provides health care and LTSS staff more tools to use when attempting to improve the mental health and well-being of an older adult in their care.

It is also important to note that the needs analyzed in this study (supportive equipment, transportation, and social activities), while overall having similar relationships with depressive symptoms, showed different patterns of need and the frequency in which the needs were met. For example, similar numbers of LTSS recipients reported a new transportation need 3 months later (11%) as those who reported their transportation need being met (12%, Table 2). It is possible that transportation needs have a high degree of variability, indicating that it would be valuable to continue to reassess this need. Supportive equipment needs, on the other hand, had the lowest level of new needs reported (8%) compared with transportation and social activities needs. It is possible that new supportive equipment needs develop more slowly (e.g., deteriorating hearing or eyesight over time) or occur in response to major events that cause a new functional impairment (e.g., a fall, stroke, or surgery). The supportive equipment needs also had the highest levels of no need at baseline (70%) and after 3 months (75%). Therefore, an effective strategy for this need may be to focus on assessment and reassessment of the highest risk groups, such as those who have had a recent change in health status or those with mild sensory impairment. Social activities were the only need of the three asked about that was higher 3 months later. This was driven by the smallest number of LTSS recipients indicating that their baseline social activities need was met after 3 months (10%), and the highest number of LTSS recipients indicating that they had a new need after 3 months that was not present at baseline (13%). It is possible that as an individual acclimates to their new LTSS setting or support they have a stronger feeling of missing social activities that they used to participate in but are no longer available or accessible. The provision of more diverse activity options based on LTSS recipient feedback may have an impact on this disparity.

### Limitations

There were some limitations to this study and the data set. All of the needs data were self-report, based on the perception of each LTSS recipient. Data on what it would mean to “meet” a given need (e.g., transportation on demand vs. limited access to transportation) or how much “meeting” that need meant to the older adult (e.g., supportive equipment gave them access to new independent experiences) was not asked in the parent study. In addition, the social activities need question (“Do you feel there are enough activities that you like available to you now?”) was framed differently than the other two questions that were explicitly framed in terms of a need. This information could provide clues to determine the mechanism behind the relationship between needs and depressive symptoms. Another limitation is the inability to include race or ethnicity in the analyses due to the imbalance of race and ethnicity proportions in the different LTSS settings. It is indeed possible that the basis for the strength and direction of the relationship between LTSS type and depressive symptoms in our regression analyses could be due to race and ethnicity differences concealed within the LTSS variable. Future research that can control for race and ethnicity across LTSS types is needed.

Similarly, our subset had fewer individuals from the NH LTSS setting than ALCs or those receiving HCBS due to missing data at the 3-month follow-up. Findings from the parent study examining hospitalizations and other types of health care resource use found that LTSS recipients in NHs had the greatest number of resource use events at 3 months compared with LTSS recipients in ALCs or receiving services from home- and community-based organizations (Hirschman, Toles, Hanlon, Huang, & Naylor, 2019). It is likely that the NH LTSS recipients who missed their follow-up interview after 3 months were hospitalized. An additional limitation was that the exact timing of the follow-up interviews (time from the start of services) varied for each participant. While the participant was enrolled, and the “baseline” interview completed within 60 days of LTSS enrollment, this average was closer to 44 days (median: 42). As there was variability, these data are likely capturing participants’ perspectives at 4–6 months following the initial interview. For our regression analyses, though we identified a statistically significant difference in GDS-SF scores, it is difficult to establish whether the difference was clinically significant. However, we believe that any changes in the number of depressive symptoms are important to monitor. Finally, we cannot make any causal inferences based on the available data and analysis options.

Future research should expand on these results with more in-depth or open-ended qualitative questions about what having these needs met would give to an older adult in LTSS settings and how that might improve mood. Clinicians working with the older adult LTSS population should prioritize asking about patients’ needs and identify available resources to meet those needs.

## Funding

This work was supported by the National Institute on Aging [R01AG025524 to M.D. Naylor] and the National Institute of Nursing Research [T32NR009356 to M. D. Naylor].

## Conflict of Interest

None reported.
